# Cost of Atrial Fibrillation: Invasive vs Non-Invasive Management in 2012

**DOI:** 10.2174/157340312803760730

**Published:** 2012-11

**Authors:** Yaariv Khaykin, Yana Shamiss

**Affiliations:** Heart Rhythm Program, Southlake Regional Health Centre, 105-712 Davis Drive, Newmarket, Ontario, L3Y 8C3, Canada

**Keywords:** Atrial fibrillation, catheter ablation, cost-effectiveness, aniarrhythmic therapy, antithrombotic therapy.

## Abstract

Atrial fibrillation (AF) is the most common sustained cardiac arrhythmia. It places an enormous burden on the patients, caregivers and the society at large. As a chronic illness, AF accrues significant costs related to clinical presentation, complications and loss of productivity. Novel invasive approaches to AF promise a cure in some patients and a significant reduction in AF burden in others, but are very expensive. This paper will address the cost of conventional and invasive strategies in AF care and will review the evidence on the comparative cost effectiveness of these approaches.

## INTRODUCTION

Atrial fibrillation (AF) is responsible for most arrhythmia related hospital admissions [1] and is the most common cause of ischemic stroke [2]. Furthermore, AF carries a tremendous negative impact on the quality of life and is associated with increased mortality [3]. Its prevalence is rising in our ageing society [4, 5] and so does the expense related to its management [6] and productivity lost among the suffering patients [7].

AF related complications and disability as well as AF treatment strategies contribute to a tremendous cost to the healthcare system and the society at large with system cost attributable to AF of over 2 billion US dollars spent only on the care of patients with AF-related strokes in the US Medicare system, and a total estimated medical expenditure related to AF around 6.5 billion US dollars per year [8].

The exact economic burden of AF is hard to define. While a systematic review of AF-related costs revealed that the overall average annual cost to manage one AF patient is $7,015 in 2010 US dollars [8], two other studies estimated the entire annual system cost for all care for patients with AF to range between $20,613 to $40,169. Hospitalizations are the most important determinant of the total cost (58%) with the cost of a single acute admission in Ontario with AF as a primary diagnosis of $23,392 in 2010 US dollars [9]. Similarly, direct costs attributable to AF in the US, based on the findings from an insurance database, were $15,553 per year in 2002 with 75% of the cost related to in-patient care [8, 10]. Each AF related hospitalization in another group of Medicare insured patients cost an average of $11,085 US dollars (2004-07) [11] with each AF recurrence adding $1,600 to the bill. The Euro Heart Survey on AF published estimated annual costs of AF care ranging from €698 in Poland to €1,544 in the Netherlands in 2006 [12] Treatment costs associated with follow-up of AF patients including hospital admissions, emergency room visits, testing and follow-up with cardiologists, internists and family physicians were also reported in France [13]. This analysis stratified patients according to therapeutic strategy – rate or rhythm control – as well as according to concomitant congestive heart failure symptoms. The authors estimated the average total 5-year cost of AF at €16,539 in 1998 currency. In a recent analysis of a German insurance database, close to 80% of the cost of care in the first year following an AF related hospitalization was due to the index event with 15% attributable to the cost of drugs, and 3% to the outpatient care [7]. The cost of non-traditional adjuvants and remedies as well as that of sickness benefits, typically not included in other AF cost analyses, was on par with the cost of outpatient care – contributing about 4% of the overall treatment cost, which came in at an astounding €7,688±954 per patient in 2005 currency. 

Non-invasive therapeutic strategies for AF address restoration and maintenance of sinus rhythm, control of the ventricular rate and antithrombotic strategies directed at prevention of strokes and other embolic events. 

While historically invasive therapy for AF involved primarily elimination of AV nodal conduction and right ventricular pacing, this strategy is now reserved for only a minority of patients where AF cannot be managed by other means. Another invasive strategy aims to minimize the risk of embolic events and involves mechanical elimination or closure of the left atrial appendage (LAA), the area where clots related to atrial fibrillation most commonly form. Techniques for LAA closure or excision have been initially developed by the cardiac surgeons [14]. Novel LAA closure devices have recently shown promise in reducing the risk of stroke in patients who cannot take antithrombotic agents and can be placed percutaneously [15]. Finally, AF ablation targeting the pulmonary veins, responsible for most AF episodes and other special regions thought to promote the arrhythmia has become the mainstream invasive approach to the management of this condition. This review will address the comparative cost effectiveness of AF ablation against that of conservative care.

## CONVENTIONAL THERAPY

### Preventing Embolic Sequelae

Prevention of embolic complications is the most important aspect of care for AF patients. These range from transient ischemic events (TIA) to strokes and are the most costly complication of atrial fibrillation. Strokes secondary to AF are more severe than those secondary to atherosclerotic disease and impart a greater disability on the victims [6]. This results in significant costs related to hospitalizations, rehabilitation and chronic disability. Strategies aimed at reducing embolic events in AF patients include therapy with aspirin, combination of aspirin and clopidogrel, and oral anticoagulation therapy with warfarin or one of the new agents targeting either thrombin or Factor IIa [16-18]. In the study of Dagibatran vs Warfarin in Patients with Atrial Fibrillation (RE-LY) the use of dabigatran, a direct thrombin inhibitor, was associated with similar rates of stroke and systemic embolism but lower rate of major bleeding compared to warfarin at a lower dose of 110 mg, while the higher dose of the drug at 150 mg was associated with lower rates of stroke and systemic embolism but similar rates of major bleeding compared to warfarin [18]. Similarly, in the study of Apixaban vs Warfarin in Patients with Atrial Fibrillation (ARISTOTLE), apixaban, a factor Xa inhibitor was superior to warfarin in preventing stroke or systemic embolism, caused less bleeding, and resulted in lower mortality [19], while rivaroxaban in the Rivaroxaban Once-daily oral direct factor Xa inhibition Compared with vitamin K antagonism for prevention of stroke and Embolism Trial in Atrial Fibrillation (ROCKET-AF) study was shown to be non-inferior to warfarin in patients at the highest risk of stroke [20].

Several studies have addressed the cost of anticoagulation therapies and their complications. A review focusing on the cost of warfarin therapy and taking into account both cost savings in the form of embolic prevention and the expense attributable to the complications associated with this therapy estimated the annual cost of anticoagulation at $1,585.57 Canadian in 2005 [21]. Novel antithrombotic agents have been shown to further reduce the incidence of stroke and systemic embolism as well as that of major and particularly intracranial bleeding compared to warfarin. These agents do not require monitoring but carry a greater upfront cost. Several studies evaluated cost effectiveness of these therapies compared to warfarin. Shah and colleagues found dabigatran, a direct thrombin inhibitor cost effective compared to warfarin at a higher dose of 150 mg twice per day based on the findings of the Randomized Evaluation of Long Term Anticoagulation Therapy (RE-LY) study using Markov analysis [22]. In their model this finding held true among patients at a moderate to high risk of stroke in whom INR could be maintained in the therapeutic range less than 72% of the time. Their findings were supported by Pink and colleagues in a similar analysis in the UK context [23]. There has not been a systematic cost-effectiveness evaluation of the surgical left atrial appendageal exclusion or its percutaneous occlusion compared to conventional therapy.

### Control of Rate and Rhythm

From the outset of clinical investigation into AF management it made common sense to pursue normal sinus rhythm as the goal for most patients. It seemed only natural that patients in sinus rhythm should fare better than those in AF. A number of studies set out to compare outcomes in patients treated with the goal to achieve sinus rhythm or remain in atrial fibrillation with a controlled ventricular response. As a surprise to many, these studies uniformly showed little advantage to the strategy of rhythm maintenance [24-27]. Patients who were able to achieve and maintain sinus rhythm, regardless of therapeutic strategy assignment had more favorable outcomes than those who stayed in atrial fibrillation [28, 29], however, all evidence pointed to the greater cost-effectiveness of rate control driven largely by higher hospitalization rate among patients treated with the rhythm control strategy [30-32].

Dronedarone, a novel antiarrhythmic agent developed on the basis of the amiodarone molecule [33] received special attention with the Effect of Dronedarone on Cardiovascular Events in Atrial Fibrillation (ATHENA) study showing significant reduction in AF related hospitalizations with a hazard ratio of 0.626 compared to placebo. Dronedarone also reduced duration of hospitalization and the risk of stroke in these patients by close to 40%. Both of these effects would potentially reduce the cost of care by approximately $3000-6000/year based on the US and Canadian data. Cost effectiveness analysis of dronedarone was submitted for evaluation to the British National Institute for Health and Clinical Excellence (NICE). In this analysis, dronedarone was cost-effective compared to the conventional therapy with the incremental cost-effectiveness ratios (ICERs) ranging from £6757 to £7890 per quality-adjusted life year (QALY) gained. Unfortunately, this estimate hinged on the assumption that dronedarone reduced mortality, an assumption now known to be false in at least two groups of patients; those with a history of congestive heart failure with or without persistent AF [34, 35].

In one Canadian analysis, the overall annual cost of medical therapy aimed at rate or rhythm control, not accounting for dronedarone, was estimated at $410.20 in 2005 Canadian dollars [36]. A more recent cost effectiveness analysis compared AF ablation to therapy with the most effective antiarrhythmic agent, amiodarone using a similar annual cost estimate of $433.29 in 2010 Canadian dollars [37]. Incorporating the cost of anticoagulation management with warfarin, and those of rate and rhythm control therapy and follow-up care including hospitalization, the cost of medical therapy for AF was estimated at $4,840 (range $4,176–$5,060) per patient per year in 2005 Canadian dollars [36]; a figure similar to the US estimate based on the data from the FRACTAL registry of $4,000–$5,000 in annual direct healthcare costs expressed in 2002 US dollars [38]. The more recent Canadian model arrived at a similar estimate, close to $4,000 dollars in 2010 currency [37]. This latter model took into account the costs associated with amiodarone toxicity and stroke but not the cost of AF follow-up among the medically treated patients.

## AF ABLATION

### Surgical Approach

The advent of catheter ablation for atrial fibrillation has come on the heels of AF surgery introduced by Dr. J Cox in 1987. The Cox-MAZE procedure was shown to successfully restore and maintain sinus rhythm [39, 40]. It has undergone several iterations and has morphed from the traditional ‘cut-and-saw’ procedure to ablation using radiofrequency, cryo, microwave or high frequency ultrasound energy targeting the pulmonary veins and the posterior left atrium with addition of linear ablation in the right and left atria depending on patient presentation. Surgical ablation has been studied as a stand-alone procedure as well as an addition to other heart surgery, typically involving the mitral valve. This latter application is responsible for most of these procedures to date and has been shown to be both effective and safe apart from a greater requirement for permanent pacing following surgical ablation for AF [41]. Cost-effectiveness analysis of six randomized controlled trials of mitral valve surgery with concomitant surgical AF ablation, modeling effectiveness in terms of freedom from atrial fibrillation demonstrated this to be a safe and effective approach with incremental cost effectiveness of $4,446 Canadian (2009) over mitral valve surgery alone and 74% likelihood of cost-effectiveness at the traditional willingness to pay $50,000.00 per QALY gained [42]. Conversely, a cost effectiveness analysis performed as part of a randomized trial of additional surgical AF ablation in addition to other heart surgery in 150 patients showed little difference in QALY gained using either approach with incremental cost effectiveness of €73,359 per QALY (2011), making it a not cost-effective [43].

### Percutaneous Catheter Ablation

Catheter ablation has rapidly moved to the mainstream of AF therapy. This approach is based on the notion that paroxysmal AF episodes arise as a result of focal firing in the pulmonary veins and elsewhere in the left and right atria [44]. While initially considered ‘curative’, over the last few years it is becoming apparent that many patients treated with catheter ablation return with further episodes of arrhythmia down the road [45], and many more continue to experience asymptomatic episodes of arrhythmia [46]. Nevertheless, studies of AF ablation have uniformly found improved quality of life among ablated patients. Some of these studies have also demonstrated a significant reduction in resource utilization [47] following ablation as well as a reduction in the risk of stroke and mortality [48].

Several studies estimated the cost of the catheter ablation procedure [36, 49, 50]. Cost of ablation typically accounts for the use of hospital resources, catheters, physician fees, associated tests and complications of the procedure. Since many patients may require further ablation due to downstream arrhythmia recurrences, the costs associated with these have to be factored in as well. In an analysis of Medicare patients followed for a year after ablation, Kim *et al*. found the cost of successful ablation at US$16,049 ± 12,536 versus US$19,997 ± 13,958 for failed ablation with 51% ablation success rate in this cohort [49]. In the Canadian publication, the overall cost of ablation was estimated at $16,278 to $21,294 in 2005 Canadian dollars, taking all of these factors into account, with the cost of ablation itself ~$9000 [36], a figure similar to the estimate used in the Canadian Agency for Drugs and Technologies in Health (CADTH) document of $9,590 [37]. 

## COST-EFFECTIVENESS PERSPECTIVE

Several projections of cost of care of an AF patient have been published in an attempt to estimate the relative cost of ablation and contrast it to the cost of medical therapy over time [36, 50]. A study directly comparing the costs of ablation and medical therapy in the Canadian healthcare environment has been published [36]. Costs related to medical therapy in the analysis included the cost of anticoagulation, rate and rhythm control medications, non-invasive testing, physician follow-up visits and hospital admissions, as well as the cost of complications related to this management strategy. Costs related to catheter ablation were assumed to include the cost of the ablation tools (electroanatomic mapping or intracardiac echocardiography-guided pulmonary vein ablation), hospital and physician billings, costs related to periprocedural medical care and complications. Costs related to these various elements were obtained from the Canadian Registry of Atrial Fibrillation (CARAF), government fee schedules and published data. Sensitivity analyses looking at a range of initial success rates (50-75%) and late attrition rates (1-5%), prevalence of congestive heart failure (20-60%) as well as discounting varying from 3 to 5% per year were performed. In this study, the cost of catheter ablation strategy ranged from ~US$14,000 to US$18,000. It was assumed that patients who required anticoagulation prior to ablation would continue on this therapy following the procedure with an annual average follow-up cost of US$1400 to US$1800 among the ablated patients. The annual cost of medical therapy ranged from US$3,600 to US$4300. The study projected costs of ongoing medical therapy and catheter ablation to equalize at 3.2 to 8.4 years of follow-up in this study but did not take into account development of the novel antiarrhythmic and thromboprophylactic strategies not available at the time of the publication nor the lower long term sinus rhythm maintenance rates, which had been reported since.

Six groups of investigators attempted to perform a cost-benefit analysis of AF ablation with that of medical therapy [37, 51-55]. In the first of these studies, a Markov decision analysis model looking at 55 and 65-year-old cohorts of patients at low and moderate risk of stroke was created by the investigators [51]. Complications and costs related to AF, medical therapy and catheter ablation were accounted for. The model assumed that amiodarone would be used for rhythm control and a combination of digoxin and atenolol – for rate control. Eighty percent efficacy of AF ablation was assumed with 30% redo rate during the first year and 2% per year late success attrition rate. It was further assumed that as many as 38% of the patients on rate control would convert to sinus rhythm with annual AF relapse rate of 5%. Moderate risk of stroke was defined as having one risk factor, including diabetes, hypertension, coronary artery disease, or congestive heart failure. Patients at low risk of stroke were assumed to have no such risk factors. For the purpose of the model, patients at moderate risk of stroke were anticoagulated whereas those at low risk could be on warfarin or aspirin. The model incorporated annual stroke risk of 2.3% and 1.1% for patients treated with aspirin and 1.3% and 0.7% for those on warfarin at moderate and low risk for stroke respectively. A relative stroke risk of 1.4% per decade was accounted for. Age adjusted mortality based on life tables and mortality reductions attributable to aspirin and warfarin were accounted for. All health care costs were calculated in 2004 US dollars using 3% discounting per year. Costs were estimated based on Medicare reimbursement rates, hospital accounting information, published literature and the Red Book for wholesale drug costs. Catheter ablation appeared to be most cost-effective in younger patients at moderate risk of stroke at $28,700/QALY gained. It was somewhat less cost effective in the older moderate risk patients at $51,800/QALY gained and least cost-effective among the younger patients at low risk of stroke at $98,900/ QALY gained. Unfortunately, while there is no prospective data on the efficacy of ablation for prevention of thromboembolic events, the findings of this study are conditional on such evidence coming to light in the years to come. 

Eckard *et al.* developed a decision-analytic model to estimate costs, health outcomes and incremental cost-effectiveness of radiofrequency ablation compared to antiarrhythmic drug therapy for AF with a lifetime time horizon [52]. The authors used a decision tree for the initial year in which the RFA procedure is assumed to take place, and a long-term Markov structure for subsequent years. The authors factored in the potential for a second ablation within a year of the first procedure in patients still suffering from AF. They assumed 70-80% ablation success within the first year with 1.4 ablations per patient required to maintain rhythm based on Swedish data. The cost of ablation was estimated at around US$12,000, including the cost of 3-4 days in hospital, all diagnostic examinations necessary as well as the cost of disposables. Annual cost of AF therapy was estimated at US$2000. In order to estimate QALY weights for different health states, age-adjusted QALY weights based on a Swedish general population were applied for patients in the controlled AF state, and used as reference points. A decrement of 0.1 for uncontrolled AF and 0.25 for stroke was applied to the baseline utility in the controlled AF state. With annual success attrition rates of 5%, 10% and 15% used in the sensitivity analysis, the relative cost of ablation was estimated up to US$58000 per QALY without assuming stroke prevention related to the ablation strategy. 

A similar analysis in the United Kingdom suggested incremental cost effectiveness of ablation at US$16,000 per QALY in 2008 dollars [53]. The authors of this paper assumed freedom from AF at 84% at one year with 2-4%/year rate of success attrition over time resulting in their estimates favouring ablation over the other published economic analyses. Further sensitivity analyses found the estimate to depend significantly both on the relative QOL estimate associated with sinus rhythm and on the prognostic implications of being in rhythm [53].

Reynolds and his group published a Markov model cost effectiveness analysis of ablation vs antiarhrtyhmic therapy in a simulated cohort of patients with paroxysmal drug refractory AF projected over 5 years [54]. The authors assumed 60% success of the ablation approach with a 25% rate of repeat ablation. Utilities for QOL assessment were derived from real-life data, using the FRACTAL registry for the medically treated patients which collected SF-12 scores and patients ablated at the authors’ institution as well as those enrolled in the A4 trial for derivation of the scores in this cohort based on the SF-36 questionnaire. In the base scenario, the incremental cost per QALY among ablated patients was US$47,333 with cost neutrality achieved at ~10 years [54]. 

A more recent Canadian cost-effectiveness analysis of 2^nd^ line AF ablation modeled outcomes for a 65 year old male with paroxysmal AF and CHADS-2 score of 2 [37]. Amiodarone therapy was used as control. The model comprised a one-year decision tree and a longer-term Markov model using 3-month cycles with 5% discounting. Utility estimates in the model were based on the work published by Reynolds *et al*. [54]. It was assumed that patients in sinus rhythm would be at a lower risk of stroke compared to those in atrial fibrillation. Investigators also assumed that successfully ablated patients would discontinue anticoagulation. Ablated patients were assumed to undergo 1.27 ablations per patient and have a fixed cost of follow-up of $666 per year. One-year success of antiarrhythmic therapy was estimated at 26% compared to 76% for ablated patients. Cost of medical care was comprised of the cost of amiodarone therapy, warfarin therapy and monitoring. It accounted for the cost of strokes and major bleeding as well as pulmonary toxicity. Cost of ablation accounted for the risk of procedural complications. The investigators found the incremental cost effectiveness of AF ablation compared to anti-arrhythmic medication to be $59,194 per quality adjusted life year (QALY) in their base case scenario. This estimate was very sensitive to improvement in the quality of life associated with sinus rhythm ($221,839 per QALY if this were not true) and to a lesser extent on sinus rhythm associated with a lesser risk of stroke ($86,129 per QALY if this were not true). Based on the model, AF ablation would become cost-effective after 5 years of follow-up. The findings supported earlier publications from our group ranging the break-even point for the cost of AF ablation and medical therapy at 3.2–8.4 years [36] and a recent Japanese analysis, placing the cross over of the costs between 3.8-14.3 years [55].

All of these models support cost effectiveness of AF ablation in older patients at a moderate risk of stroke with similar derived ICERs despite slightly different methodology. At the same time, most patients ablated to-date have been younger with a very low risk of stroke, but a significant impairment in the quality of life associated with AF. None of the models accounted for the use of novel antithrombotic medications nor for the significant late success attrition rates among ablated patients. Detailed cost studies should be tied to prospective investigation of the outcomes in the studies of atrial fibrillation management as new therapeutic agents and invasive technology become available.

## CONCLUSIONS

Atrial fibrillation is a common condition with numerous clinical implications. Medical and invasive strategies for this condition are evolving rapidly. While more expensive up front, ablation appears to be a cost-effective alternative to the non-invasive AF treatment strategies after a 3-5 year time horizon. Future studies comparing clinical outcomes in patients treated using ablation or medical therapy should collect detailed cost data at the patient level to enable a more precise cost-effectiveness analysis. 

## ABBREVIATIONS

AF: = Atrial fibrillation
LA: = Left atrium
PV: = Pulmonary vein
PVAI: = Pulmonary vein antrum isolation
QOL: = Quality of life
OAC: = Oral Anticoagulation
TIA: = Transient Ischemic Attack
CHF: = Congestive Heart Failure
INR: = International Normalized Ratio
